# Editorial: Cell polarity in a complex environment

**DOI:** 10.3389/fcell.2023.1329874

**Published:** 2023-11-13

**Authors:** Yi Wu, Bo Dong, Benjamin C. Lin

**Affiliations:** ^1^ Richard D. Berlin Center for Cell Analysis & Modeling, UConn Health, Farmington, CT, United States; ^2^ MoE Key Laboratory of Marine Genetics and Breeding, College of Marine Life Sciences, Ocean University of China, Qingdao, China; ^3^ Department of Biochemistry and Cell Biology, Stony Brook University, Stony Brook, NY, United States

**Keywords:** polarity, membrane curvature, asymmetric cell division (ACD), unc-5 netrin receptor B (UNC5B), Hu li tai shao

Cell polarity plays a crucial role in spatially segregating signaling within cells, and it is vital for various cellular functions, including motility, barrier formation, and determining cell fate. Over several decades of research, numerous signaling circuits and network principles have been uncovered, contributing to our understanding of how polarity is established in various cell systems. However, ongoing research continues to reveal new mechanisms and factors that contribute to this process. Building on the success of the first volume, “*Establishing and Maintaining Cell Polarity*,” we are pleased to present volume II of this Research Topic, which reflects the recent progress made in the field.

Historically, much of our knowledge of cell migration has been gained from using 2D experimental systems. In this volume, Pawluchin and Galic revisit the questions of how cell motion is accomplished in a 3D microenvironment, and to what degree observations acquired in a planar system can be translated into 3D. They discuss the commonalities and key differences between 2D and 3D in the context of the molecular mechanisms by which cell motion is generated in a planar system, as well as how the motion patterns of the cell emerge at the macro scale. While focusing on the cell leading edge where polarity is developed, they also provide an in-depth discussion of membrane curvature-dependent regulation of actin dynamics and signaling in both 2D and 3D.

To recapitulate the complex fibrous architecture of the extracellular matrix (ECM), growing studies of cell migration are performed using synthetic fibrous networks designed to mimic *in vivo* ECM. Such an approach may allow us to identify the topological features that dictate cell migration patterns and determine the underlying mechanisms that regulate topography-sensing in health and disease. In the current volume, Loesel et al. contribute a method article that describes how they quantify cell migration dynamics, including speed, directionality, and the number of detached cells from cell spheroids seeded onto engineered randomly oriented and aligned ECM mimicking fibers.

An interesting example of cell polarity is asymmetric cell division (ACD), which allows stem cells to generate differentiating progeny while simultaneously maintaining their own pluripotent state. In *Drosophila* neural stem cells (neuroblasts; NBs), ACD is accomplished by reorienting the mitotic spindle along the apical-basal polarity axis through a variety of conserved complexes, including the apical, mutually exclusive Partner of Inscuteable (Pins)-Mushroom body defect (Mud) and Pins-Inscuteable (Insc) complexes. Parra et al. identify Hu li tai shao (Hts; human Adducin) as a direct Mud-binding protein that may segregate Pins from Insc by inducing phase separation of the Pins-Mud complex during spindle positioning.

Another notable function of cell polarity is in axon guidance. In the developing nervous system, axon guidance relies on a variety of extracellular cues interpreted by specific receptors. In *Caenorhabditis elegans*, UNC-6/Netrin is a conserved bi-functional guidance cue that regulates dorsal-ventral axon guidance. The UNC-5 receptor was thought to maintain dorsal protrusion polarity and inhibit ventral growth cone protrusion, resulting in net dorsal growth cone advance. Mahadik and Lundquist demonstrate a novel role for a short isoform of UNC-5 (UNC-5B), which promotes dorsal polarity of growth cone filopodial protrusion and stimulates growth cone protrusion, in contrast to the previously described role of UNC-5 long in inhibiting growth cone protrusion.

While this volume is relatively brief in its coverage of this Research Topic, we would like to express our gratitude to the authors and reviewers for their dedicated contributions. We believe that these studies offer valuable insights into the field of cell polarity, particularly through the perspective of model organisms, and address less-explored aspects such as the regulation of phase separation and multi-cue interpretation.

